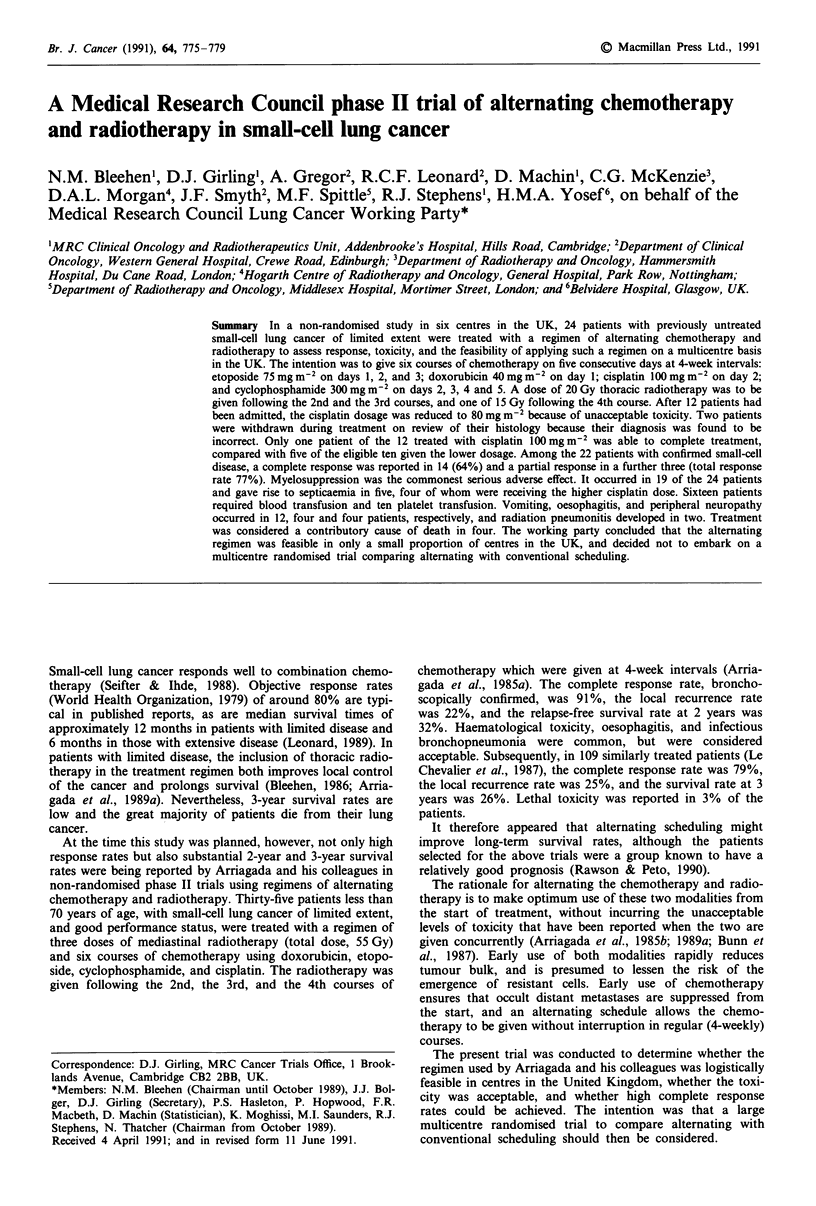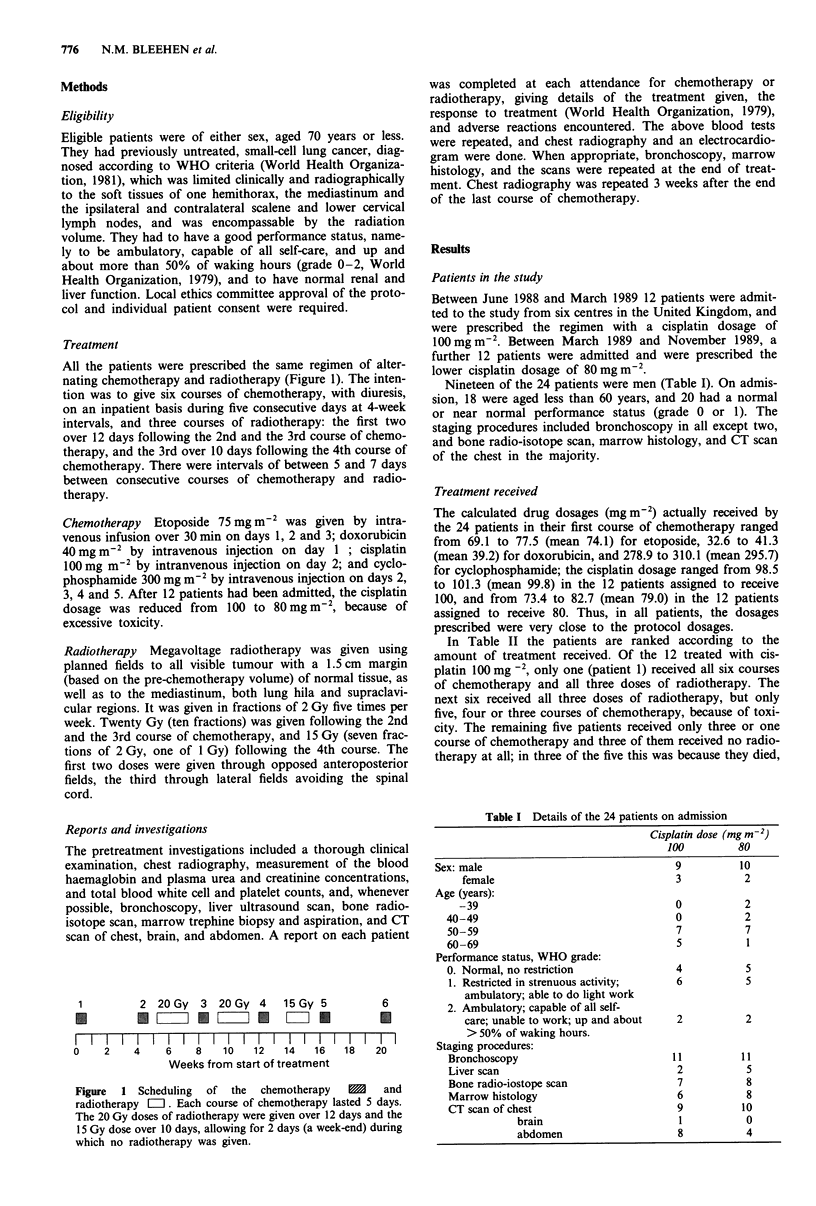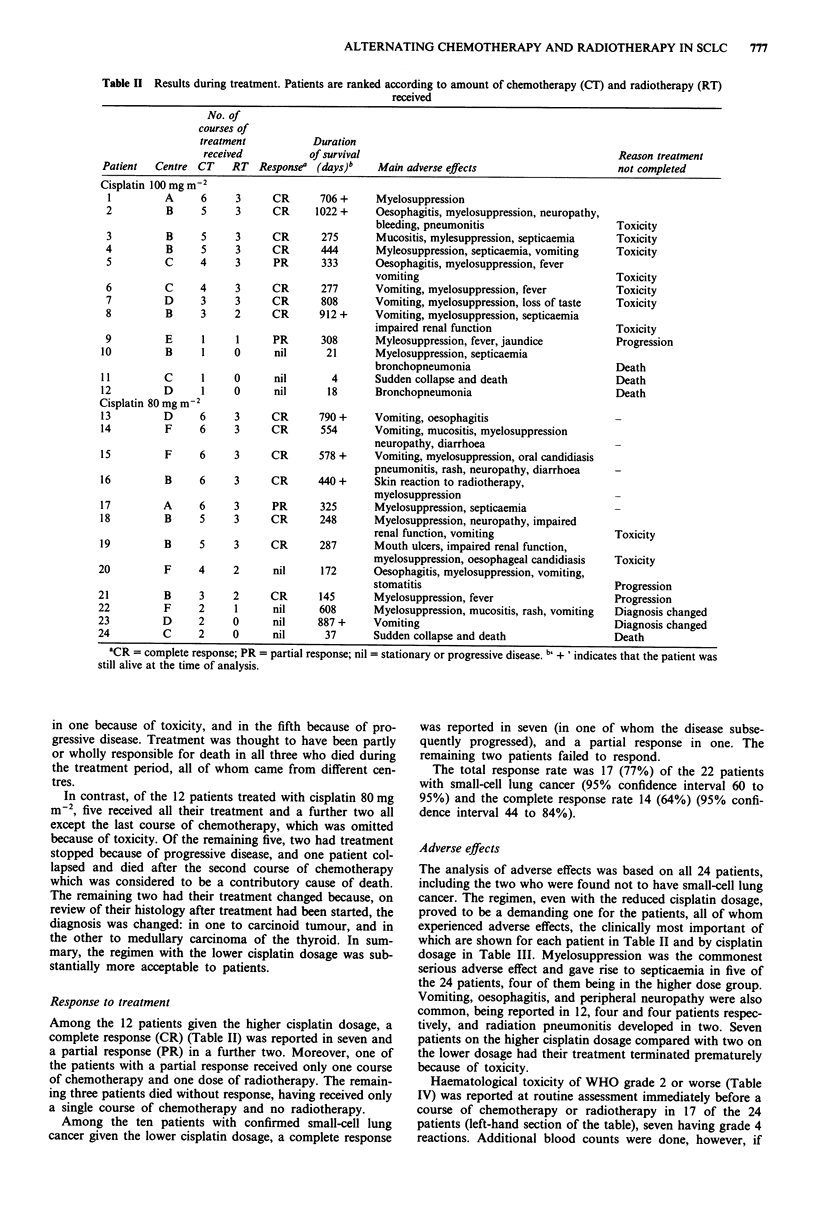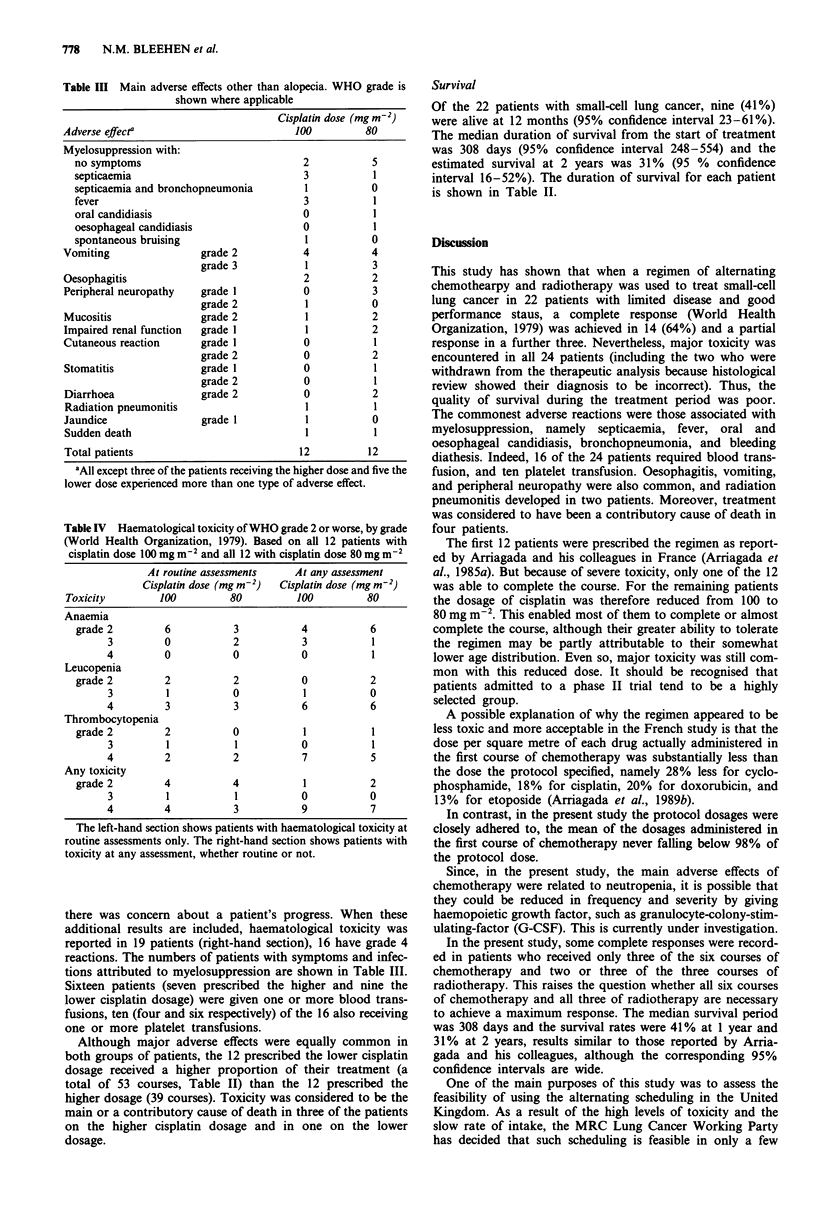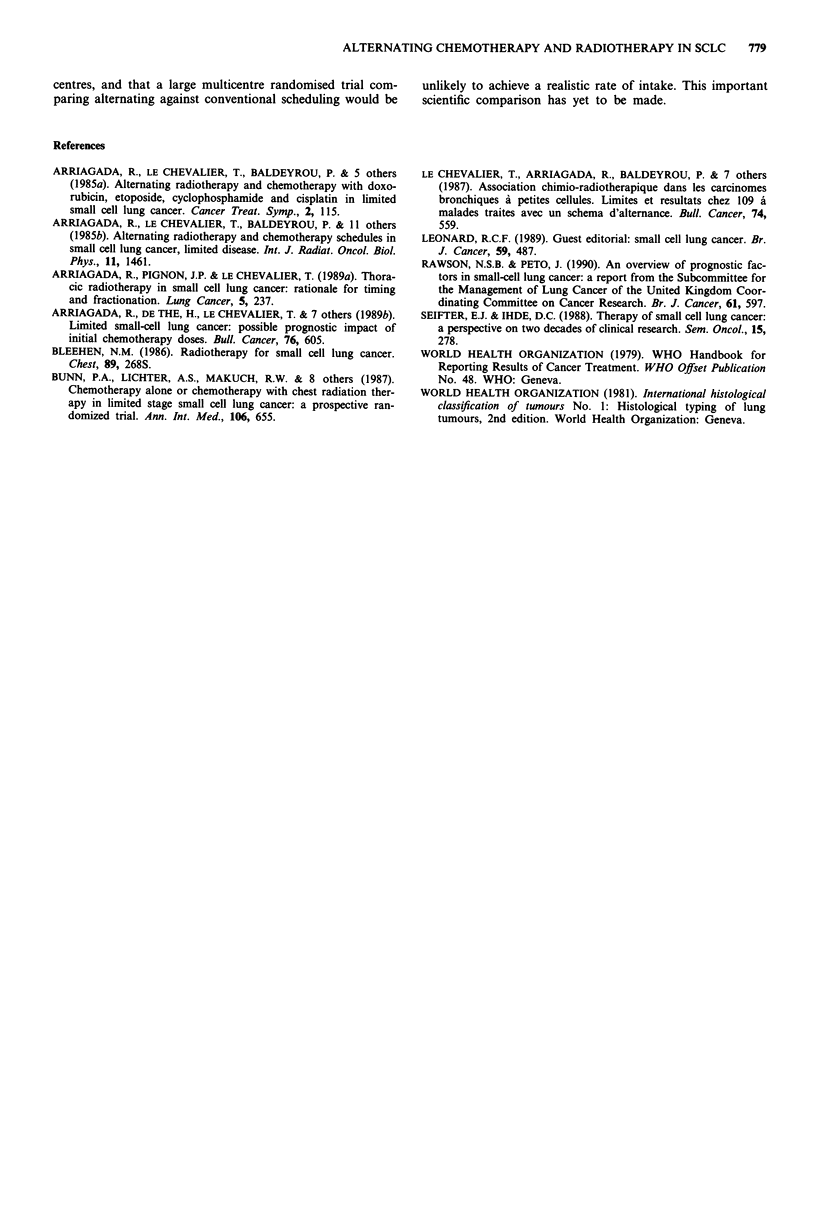# A Medical Research Council phase II trial of alternating chemotherapy and radiotherapy in small-cell lung cancer. The Medical Research Council Lung Cancer Working Party.

**DOI:** 10.1038/bjc.1991.397

**Published:** 1991-10

**Authors:** N. M. Bleehen, D. J. Girling, A. Gregor, R. C. Leonard, D. Machin, C. G. McKenzie, D. A. Morgan, J. F. Smyth, M. F. Spittle, R. J. Stephens

**Affiliations:** MRC Clinical Oncology and Radiotherapeutics Unit, Addenbrooke's Hospital, Cambridge, UK.

## Abstract

In a non-randomised study in six centres in the UK, 24 patients with previously untreated small-cell lung cancer of limited extent were treated with a regimen of alternating chemotherapy and radiotherapy to assess response, toxicity, and the feasibility of applying such a regimen on a multicentre basis in the UK. The intention was to give six courses of chemotherapy on five consecutive days at 4-week intervals: etoposide 75 mg m-2 on days 1, 2, and 3; doxorubicin 40 mg m-2 on day 1; cisplatin 100 mg m-2 on day 2; and cyclophosphamide 300 mg m-2 on days 2, 3, 4 and 5. A dose of 20 Gy thoracic radiotherapy was to be given following the 2nd and the 3rd courses, and one of 15 Gy following the 4th course. After 12 patients had been admitted, the cisplatin dosage was reduced to 80 mg m-2 because of unacceptable toxicity. Two patients were withdrawn during treatment on review of their histology because their diagnosis was found to be incorrect. Only one patient of the 12 treated with cisplatin 100 mg m-2 was able to complete treatment, compared with five of the eligible ten given the lower dosage. Among the 22 patients with confirmed small-cell disease, a complete response was reported in 14 (64%) and a partial response in a further three (total response rate 77%). Myelosuppression was the commonest serious adverse effect. It occurred in 19 of the 24 patients and gave rise to septicaemia in five, four of whom were receiving the higher cisplatin dose. Sixteen patients required blood transfusion and ten platelet transfusion. Vomiting, oesophagitis, and peripheral neuropathy occurred in 12, four and four patients, respectively, and radiation pneumonitis developed in two. Treatment was considered a contributory cause of death in four. The working party concluded that the alternating regimen was feasible in only a small proportion of centres in the UK, and decided not to embark on a multicentre randomised trial comparing alternating with conventional scheduling.


Br. J. Cancer (1991), 64, 775–779 © Macmillan Press Ltd., 1991

### A Medical Research Council phase II trial of alternating chemotherapy and radiotherapy in small-cell lung cancer

N.M. Bleehen[1], D.J. Girling[1], A. Gregor[2], R.C.F. Leonard[2], D. Machin[1], C.G. McKenzie[3], D.A.L. Morgan[4], J.F. Smyth[2], M.F. Spittle[5], R.J. Stephens[1], H.M.A. Yosef[6], on behalf of the Medical Research Council Lung Cancer Working Party*

[1]*MRC Clinical Oncology and Radiotherapeutics Unit, Addenbrooke's Hospital, Hills Road, Cambridge;* [2]*Department of Clinical Oncology, Western General Hospital, Crewe Road, Edinburgh;* [3]*Department of Radiotherapy and Oncology, Hammersmith Hospital, Du Cane Road, London;* [4]*Hogarth Centre of Radiotherapy and Oncology, General Hospital, Park Row, Nottingham;* [5]*Department of Radiotherapy and Oncology, Middlesex Hospital, Mortimer Street, London; and* [6]*Belvidere Hospital, Glasgow, UK.*

**Summary** In a non-randomised study in six centres in the UK, 24 patients with previously untreated small-cell lung cancer of limited extent were treated with a regimen of alternating chemotherapy and radiotherapy to assess response, toxicity, and the feasibility of applying such a regimen on a multicentre basis in the UK. The intention was to give six courses of chemotherapy on five consecutive days at 4-week intervals: etoposide 75 mg $m^{-2}$ on days 1, 2, and 3; doxorubicin 40 mg $m^{-2}$ on day 1; cisplatin 100 mg $m^{-2}$ on day 2; and cyclophosphamide 300 mg $m^{-2}$ on days 2, 3, 4 and 5. A dose of 20 Gy thoracic radiotherapy was to be given following the 2nd and the 3rd courses, and one of 15 Gy following the 4th course. After 12 patients had been admitted, the cisplatin dosage was reduced to 80 mg $m^{-2}$ because of unacceptable toxicity. Two patients were withdrawn during treatment on review of their histology because their diagnosis was found to be incorrect. Only one patient of the 12 treated with cisplatin 100 mg $m^{-2}$ was able to complete treatment, compared with five of the eligible ten given the lower dosage. Among the 22 patients with confirmed small-cell disease, a complete response was reported in 14 (64%) and a partial response in a further three (total response rate 77%). Myelosuppression was the commonest serious adverse effect. It occurred in 19 of the 24 patients and gave rise to septicaemia in five, four of whom were receiving the higher cisplatin dose. Sixteen patients required blood transfusion and ten platelet transfusion. Vomiting, oesophagitis, and peripheral neuropathy occurred in 12, four and four patients, respectively, and radiation pneumonitis developed in two. Treatment was considered a contributory cause of death in four. The working party concluded that the alternating regimen was feasible in only a small proportion of centres in the UK, and decided not to embark on a multicentre randomised trial comparing alternating with conventional scheduling.

Small-cell lung cancer responds well to combination chemotherapy (Seifter & Ihde, 1988). Objective response rates (World Health Organization, 1979) of around 80% are typical in published reports, as are median survival times of approximately 12 months in patients with limited disease and 6 months in those with extensive disease (Leonard, 1989). In patients with limited disease, the inclusion of thoracic radiotherapy in the treatment regimen both improves local control of the cancer and prolongs survival (Bleehen, 1986; Arriagada *et al.*, 1989*a*). Nevertheless, 3-year survival rates are low and the great majority of patients die from their lung cancer.

At the time this study was planned, however, not only high response rates but also substantial 2-year and 3-year survival rates were being reported by Arriagada and his colleagues in non-randomised phase II trials using regimens of alternating chemotherapy and radiotherapy. Thirty-five patients less than 70 years of age, with small-cell lung cancer of limited extent, and good performance status, were treated with a regimen of three doses of mediastinal radiotherapy (total dose, 55 Gy) and six courses of chemotherapy using doxorubicin, etoposide, cyclophosphamide, and cisplatin. The radiotherapy was given following the 2nd, the 3rd, and the 4th courses of chemotherapy which were given at 4-week intervals (Arriagada *et al.*, 1985*a*). The complete response rate, bronchoscopically confirmed, was 91%, the local recurrence rate was 22%, and the relapse-free survival rate at 2 years was 32%. Haematological toxicity, oesophagitis, and infectious bronchopneumonia were common, but were considered acceptable. Subsequently, in 109 similarly treated patients (Le Chevalier *et al.*, 1987), the complete response rate was 79%, the local recurrence rate was 25%, and the survival rate at 3 years was 26%. Lethal toxicity was reported in 3% of the patients.

It therefore appeared that alternating scheduling might improve long-term survival rates, although the patients selected for the above trials were a group known to have a relatively good prognosis (Rawson & Peto, 1990).

The rationale for alternating the chemotherapy and radiotherapy is to make optimum use of these two modalities from the start of treatment, without incurring the unacceptable levels of toxicity that have been reported when the two are given concurrently (Arriagada *et al.*, 1985*b*; 1989*a*; Bunn *et al.*, 1987). Early use of both modalities rapidly reduces tumour bulk, and is presumed to lessen the risk of the emergence of resistant cells. Early use of chemotherapy ensures that occult distant metastases are suppressed from the start, and an alternating schedule allows the chemotherapy to be given without interruption in regular (4-weekly) courses.

The present trial was conducted to determine whether the regimen used by Arriagada and his colleagues was logistically feasible in centres in the United Kingdom, whether the toxicity was acceptable, and whether high complete response rates could be achieved. The intention was that a large multicentre randomised trial to compare alternating with conventional scheduling should then be considered.

Correspondence: D.J. Girling, MRC Cancer Trials Office, 1 Brooklands Avenue, Cambridge CB2 2BB, UK.
*Members: N.M. Bleehen (Chairman until October 1989), J.J. Bolger, D.J. Girling (Secretary), P.S. Hasleton, P. Hopwood, F.R. Macbeth, D. Machin (Statistician), K. Moghissi, M.I. Saunders, R.J. Stephens, N. Thatcher (Chairman from October 1989).
Received 4 April 1991; and in revised form 11 June 1991.

### Methods

### *Eligibility*

Eligible patients were of either sex, aged 70 years or less. They had previously untreated, small-cell lung cancer, diagnosed according to WHO criteria (World Health Organization, 1981), which was limited clinically and radiographically to the soft tissues of one hemithorax, the mediastinum and the ipsilateral and contralateral scalene and lower cervical lymph nodes, and was encompassable by the radiation volume. They had to have a good performance status, namely to be ambulatory, capable of all self-care, and up and about more than 50% of waking hours (grade 0–2, World Health Organization, 1979), and to have normal renal and liver function. Local ethics committee approval of the protocol and individual patient consent were required.

### *Treatment*

All the patients were prescribed the same regimen of alternating chemotherapy and radiotherapy (Figure 1). The intention was to give six courses of chemotherapy, with diuresis, on an inpatient basis during five consecutive days at 4-week intervals, and three courses of radiotherapy: the first two over 12 days following the 2nd and the 3rd course of chemotherapy, and the 3rd over 10 days following the 4th course of chemotherapy. There were intervals of between 5 and 7 days between consecutive courses of chemotherapy and radiotherapy.

*Chemotherapy* Etoposide 75 mg $m^{-2}$ was given by intravenous infusion over 30 min on days 1, 2 and 3; doxorubicin 40 mg $m^{-2}$ by intravenous injection on day 1 ; cisplatin 100 mg $m^{-2}$ by intranvenous injection on day 2; and cyclophosphamide 300 mg $m^{-2}$ by intravenous injection on days 2, 3, 4 and 5. After 12 patients had been admitted, the cisplatin dosage was reduced from 100 to 80 mg $m^{-2}$, because of excessive toxicity.

*Radiotherapy* Megavoltage radiotherapy was given using planned fields to all visible tumour with a 1.5 cm margin (based on the pre-chemotherapy volume) of normal tissue, as well as to the mediastinum, both lung hila and supraclavicular regions. It was given in fractions of 2 Gy five times per week. Twenty Gy (ten fractions) was given following the 2nd and the 3rd course of chemotherapy, and 15 Gy (seven fractions of 2 Gy, one of 1 Gy) following the 4th course. The first two doses were given through opposed anteroposterior fields, the third through lateral fields avoiding the spinal cord.

### *Reports and investigations*

The pretreatment investigations included a thorough clinical examination, chest radiography, measurement of the blood haemaglobin and plasma urea and creatinine concentrations, and total blood white cell and platelet counts, and, whenever possible, bronchoscopy, liver ultrasound scan, bone radioisotope scan, marrow trephine biopsy and aspiration, and CT scan of chest, brain, and abdomen. A report on each patient was completed at each attendance for chemotherapy or radiotherapy, giving details of the treatment given, the response to treatment (World Health Organization, 1979), and adverse reactions encountered. The above blood tests were repeated, and chest radiography and an electrocardiogram were done. When appropriate, bronchoscopy, marrow histology, and the scans were repeated at the end of treatment. Chest radiography was repeated 3 weeks after the end of the last course of chemotherapy.

**Figure 1** Scheduling of the chemotherapy ▨ and radiotherapy □. Each course of chemotherapy lasted 5 days. The 20 Gy doses of radiotherapy were given over 12 days and the 15 Gy dose over 10 days, allowing for 2 days (a week-end) during which no radiotherapy was given.

### Results

### *Patients in the study*

Between June 1988 and March 1989 12 patients were admitted to the study from six centres in the United Kingdom, and were prescribed the regimen with a cisplatin dosage of 100 mg $m^{-2}$. Between March 1989 and November 1989, a further 12 patients were admitted and were prescribed the lower cisplatin dosage of 80 mg $m^{-2}$.

Nineteen of the 24 patients were men (Table I). On admission, 18 were aged less than 60 years, and 20 had a normal or near normal performance status (grade 0 or 1). The staging procedures included bronchoscopy in all except two, and bone radio-isotope scan, marrow histology, and CT scan of the chest in the majority.

### *Treatment received*

The calculated drug dosages (mg $m^{-2}$) actually received by the 24 patients in their first course of chemotherapy ranged from 69.1 to 77.5 (mean 74.1) for etoposide, 32.6 to 41.3 (mean 39.2) for doxorubicin, and 278.9 to 310.1 (mean 295.7) for cyclophosphamide; the cisplatin dosage ranged from 98.5 to 101.3 (mean 99.8) in the 12 patients assigned to receive 100, and from 73.4 to 82.7 (mean 79.0) in the 12 patients assigned to receive 80. Thus, in all patients, the dosages prescribed were very close to the protocol dosages.

In Table II the patients are ranked according to the amount of treatment received. Of the 12 treated with cisplatin 100 mg $^{-2}$, only one (patient 1) received all six courses of chemotherapy and all three doses of radiotherapy. The next six received all three doses of radiotherapy, but only five, four or three courses of chemotherapy, because of toxicity. The remaining five patients received only three or one course of chemotherapy and three of them received no radiotherapy at all; in three of the five this was because they died,

**Table I** Details of the 24 patients on admission

| | *Cisplatin dose (mg $m^{-2}$)* 100 | 80 |
|---|---|---|
| Sex: male | 9 | 10 |
| female | 3 | 2 |
| Age (years): | | |
| -39 | 0 | 2 |
| 40–49 | 0 | 2 |
| 50–59 | 7 | 7 |
| 60–69 | 5 | 1 |
| Performance status, WHO grade: | | |
| 0. Normal, no restriction | 4 | 5 |
| 1. Restricted in strenuous activity; ambulatory; able to do light work | 6 | 5 |
| 2. Ambulatory; capable of all self-care; unable to work; up and about >50% of waking hours. | 2 | 2 |
| Staging procedures: | | |
| Bronchoscopy | 11 | 11 |
| Liver scan | 2 | 5 |
| Bone radio-iostope scan | 7 | 8 |
| Marrow histology | 6 | 8 |
| CT scan of chest | 9 | 10 |
| brain | 1 | 0 |
| abdomen | 8 | 4 |

**Table II** Results during treatment. Patients are ranked according to amount of chemotherapy (CT) and radiotherapy (RT) received

| *Patient* | *Centre* | *No. of courses of treatment received* CT | RT | *Response*[a] | *Duration of survival (days)*[b] | *Main adverse effects* | *Reason treatment not completed* |
|---|---|---|---|---|---|---|---|
| Cisplatin 100 mg $m^{-2}$ | | | | | | | |
| 1 | A | 6 | 3 | CR | 706+ | Myelosuppression | |
| 2 | B | 5 | 3 | CR | 1022+ | Oesophagitis, myelosuppression, neuropathy, bleeding, pneumonitis | Toxicity |
| 3 | B | 5 | 3 | CR | 275 | Mucositis, mylesuppression, septicaemia | Toxicity |
| 4 | B | 5 | 3 | CR | 444 | Myleosuppression, septicaemia, vomiting | Toxicity |
| 5 | C | 4 | 3 | PR | 333 | Oesophagitis, myelosuppression, fever vomiting | Toxicity |
| 6 | C | 4 | 3 | CR | 277 | Vomiting, myelosuppression, fever | Toxicity |
| 7 | D | 3 | 3 | CR | 808 | Vomiting, myelosuppression, loss of taste | Toxicity |
| 8 | B | 3 | 2 | CR | 912+ | Vomiting, myelosuppression, septicaemia impaired renal function | Toxicity |
| 9 | E | 1 | 1 | PR | 308 | Myleosuppression, fever, jaundice | Progression |
| 10 | B | 1 | 0 | nil | 21 | Myelosuppression, septicaemia bronchopneumonia | Death |
| 11 | C | 1 | 0 | nil | 4 | Sudden collapse and death | Death |
| 12 | D | 1 | 0 | nil | 18 | Bronchopneumonia | Death |
| Cisplatin 80 mg $m^{-2}$ | | | | | | | |
| 13 | D | 6 | 3 | CR | 790+ | Vomiting, oesophagitis | – |
| 14 | F | 6 | 3 | CR | 554 | Vomiting, mucositis, myelosuppression neuropathy, diarrhoea | – |
| 15 | F | 6 | 3 | CR | 578+ | Vomiting, myelosuppression, oral candidiasis pneumonitis, rash, neuropathy, diarrhoea | – |
| 16 | B | 6 | 3 | CR | 440+ | Skin reaction to radiotherapy, myelosuppression | – |
| 17 | A | 6 | 3 | PR | 325 | Myelosuppression, septicaemia | – |
| 18 | B | 5 | 3 | CR | 248 | Myelosuppression, neuropathy, impaired renal function, vomiting | Toxicity |
| 19 | B | 5 | 3 | CR | 287 | Mouth ulcers, impaired renal function, myelosuppression, oesophageal candidiasis | Toxicity |
| 20 | F | 4 | 2 | nil | 172 | Oesophagitis, myelosuppression, vomiting, stomatitis | Progression |
| 21 | B | 3 | 2 | CR | 145 | Myelosuppression, fever | Progression |
| 22 | F | 2 | 1 | nil | 608 | Myelosuppression, mucositis, rash, vomiting | Diagnosis changed |
| 23 | D | 2 | 0 | nil | 887+ | Vomiting | Diagnosis changed |
| 24 | C | 2 | 0 | nil | 37 | Sudden collapse and death | Death |

[a]CR = complete response; PR = partial response; nil = stationary or progressive disease. [b]' + ' indicates that the patient was still alive at the time of analysis.

in one because of toxicity, and in the fifth because of progressive disease. Treatment was thought to have been partly or wholly responsible for death in all three who died during the treatment period, all of whom came from different centres.

In contrast, of the 12 patients treated with cisplatin 80 mg $m^{-2}$, five received all their treatment and a further two all except the last course of chemotherapy, which was omitted because of toxicity. Of the remaining five, two had treatment stopped because of progressive disease, and one patient collapsed and died after the second course of chemotherapy which was considered to be a contributory cause of death. The remaining two had their treatment changed because, on review of their histology after treatment had been started, the diagnosis was changed: in one to carcinoid tumour, and in the other to medullary carcinoma of the thyroid. In summary, the regimen with the lower cisplatin dosage was substantially more acceptable to patients.

*Response to treatment*

Among the 12 patients given the higher cisplatin dosage, a complete response (CR) (Table II) was reported in seven and a partial response (PR) in a further two. Moreover, one of the patients with a partial response received only one course of chemotherapy and one dose of radiotherapy. The remaining three patients died without response, having received only a single course of chemotherapy and no radiotherapy.

Among the ten patients with confirmed small-cell lung cancer given the lower cisplatin dosage, a complete response was reported in seven (in one of whom the disease subsequently progressed), and a partial response in one. The remaining two patients failed to respond.

The total response rate was 17 (77%) of the 22 patients with small-cell lung cancer (95% confidence interval 60 to 95%) and the complete response rate 14 (64%) (95% confidence interval 44 to 84%).

*Adverse effects*

The analysis of adverse effects was based on all 24 patients, including the two who were found not to have small-cell lung cancer. The regimen, even with the reduced cisplatin dosage, proved to be a demanding one for the patients, all of whom experienced adverse effects, the clinically most important of which are shown for each patient in Table II and by cisplatin dosage in Table III. Myelosuppression was the commonest serious adverse effect and gave rise to septicaemia in five of the 24 patients, four of them being in the higher dose group. Vomiting, oesophagitis, and peripheral neuropathy were also common, being reported in 12, four and four patients respectively, and radiation pneumonitis developed in two. Seven patients on the higher cisplatin dosage compared with two on the lower dosage had their treatment terminated prematurely because of toxicity.

Haematological toxicity of WHO grade 2 or worse (Table IV) was reported at routine assessment immediately before a course of chemotherapy or radiotherapy in 17 of the 24 patients (left-hand section of the table), seven having grade 4 reactions. Additional blood counts were done, however, if

**Table III** Main adverse effects other than alopecia. WHO grade is shown where applicable

| *Adverse effect*[a] | | *Cisplatin dose (mg m$^{-2}$)* 100 | 80 |
|---|---|---|---|
| Myelosuppression with: | | | |
| no symptoms | | 2 | 5 |
| septicaemia | | 3 | 1 |
| septicaemia and bronchopneumonia | | 1 | 0 |
| fever | | 3 | 1 |
| oral candidiasis | | 0 | 1 |
| oesophageal candidiasis | | 0 | 1 |
| spontaneous bruising | | 1 | 0 |
| Vomiting | grade 2 | 4 | 4 |
| | grade 3 | 1 | 3 |
| Oesophagitis | | 2 | 2 |
| Peripheral neuropathy | grade 1 | 0 | 3 |
| | grade 2 | 1 | 0 |
| Mucositis | grade 2 | 1 | 2 |
| Impaired renal function | grade 1 | 1 | 2 |
| Cutaneous reaction | grade 1 | 0 | 1 |
| | grade 2 | 0 | 2 |
| Stomatitis | grade 1 | 0 | 1 |
| | grade 2 | 0 | 1 |
| Diarrhoea | grade 2 | 0 | 2 |
| Radiation pneumonitis | | 1 | 1 |
| Jaundice | grade 1 | 1 | 0 |
| Sudden death | | 1 | 1 |
| Total patients | | 12 | 12 |

[a]All except three of the patients receiving the higher dose and five the lower dose experienced more than one type of adverse effect.

**Table IV** Haematological toxicity of WHO grade 2 or worse, by grade (World Health Organization, 1979). Based on all 12 patients with cisplatin dose 100 mg m$^{-2}$ and all 12 with cisplatin dose 80 mg m$^{-2}$

| *Toxicity* | *At routine assessments Cisplatin dose (mg m$^{-2}$)* 100 | 80 | *At any assessment Cisplatin dose (mg m$^{-2}$)* 100 | 80 |
|---|---|---|---|---|
| Anaemia | | | | |
| grade 2 | 6 | 3 | 4 | 6 |
| 3 | 0 | 2 | 3 | 1 |
| 4 | 0 | 0 | 0 | 1 |
| Leucopenia | | | | |
| grade 2 | 2 | 2 | 0 | 2 |
| 3 | 1 | 0 | 1 | 0 |
| 4 | 3 | 3 | 6 | 6 |
| Thrombocytopenia | | | | |
| grade 2 | 2 | 0 | 1 | 1 |
| 3 | 1 | 1 | 0 | 1 |
| 4 | 2 | 2 | 7 | 5 |
| Any toxicity | | | | |
| grade 2 | 4 | 4 | 1 | 2 |
| 3 | 1 | 1 | 0 | 0 |
| 4 | 4 | 3 | 9 | 7 |

The left-hand section shows patients with haematological toxicity at routine assessments only. The right-hand section shows patients with toxicity at any assessment, whether routine or not.

there was concern about a patient's progress. When these additional results are included, haematological toxicity was reported in 19 patients (right-hand section), 16 have grade 4 reactions. The numbers of patients with symptoms and infections attributed to myelosuppression are shown in Table III. Sixteen patients (seven prescribed the higher and nine the lower cisplatin dosage) were given one or more blood transfusions, ten (four and six respectively) of the 16 also receiving one or more platelet transfusions.

Although major adverse effects were equally common in both groups of patients, the 12 prescribed the lower cisplatin dosage received a higher proportion of their treatment (a total of 53 courses, Table II) than the 12 prescribed the higher dosage (39 courses). Toxicity was considered to be the main or a contributory cause of death in three of the patients on the higher cisplatin dosage and in one on the lower dosage.

*Survival*

Of the 22 patients with small-cell lung cancer, nine (41%) were alive at 12 months (95% confidence interval 23–61%). The median duration of survival from the start of treatment was 308 days (95% confidence interval 248–554) and the estimated survival at 2 years was 31% (95 % confidence interval 16–52%). The duration of survival for each patient is shown in Table II.

### Discussion

This study has shown that when a regimen of alternating chemothearpy and radiotherapy was used to treat small-cell lung cancer in 22 patients with limited disease and good performance staus, a complete response (World Health Organization, 1979) was achieved in 14 (64%) and a partial response in a further three. Nevertheless, major toxicity was encountered in all 24 patients (including the two who were withdrawn from the therapeutic analysis because histological review showed their diagnosis to be incorrect). Thus, the quality of survival during the treatment period was poor. The commonest adverse reactions were those associated with myelosuppression, namely septicaemia, fever, oral and oesophageal candidiasis, bronchopneumonia, and bleeding diathesis. Indeed, 16 of the 24 patients required blood transfusion, and ten platelet transfusion. Oesophagitis, vomiting, and peripheral neuropathy were also common, and radiation pneumonitis developed in two patients. Moreover, treatment was considered to have been a contributory cause of death in four patients.

The first 12 patients were prescribed the regimen as reported by Arriagada and his colleagues in France (Arriagada *et al.*, 1985*a*). But because of severe toxicity, only one of the 12 was able to complete the course. For the remaining patients the dosage of cisplatin was therefore reduced from 100 to 80 mg m$^{-2}$. This enabled most of them to complete or almost complete the course, although their greater ability to tolerate the regimen may be partly attributable to their somewhat lower age distribution. Even so, major toxicity was still common with this reduced dose. It should be recognised that patients admitted to a phase II trial tend to be a highly selected group.

A possible explanation of why the regimen appeared to be less toxic and more acceptable in the French study is that the dose per square metre of each drug actually administered in the first course of chemotherapy was substantially less than the dose the protocol specified, namely 28% less for cyclophosphamide, 18% for cisplatin, 20% for doxorubicin, and 13% for etoposide (Arriagada *et al.*, 1989*b*).

In contrast, in the present study the protocol dosages were closely adhered to, the mean of the dosages administered in the first course of chemotherapy never falling below 98% of the protocol dose.

Since, in the present study, the main adverse effects of chemotherapy were related to neutropenia, it is possible that they could be reduced in frequency and severity by giving haemopoietic growth factor, such as granulocyte-colony-stimulating-factor (G-CSF). This is currently under investigation.

In the present study, some complete responses were recorded in patients who received only three of the six courses of chemotherapy and two or three of the three courses of radiotherapy. This raises the question whether all six courses of chemotherapy and all three of radiotherapy are necessary to achieve a maximum response. The median survival period was 308 days and the survival rates were 41% at 1 year and 31% at 2 years, results similar to those reported by Arriagada and his colleagues, although the corresponding 95% confidence intervals are wide.

One of the main purposes of this study was to assess the feasibility of using the alternating scheduling in the United Kingdom. As a result of the high levels of toxicity and the slow rate of intake, the MRC Lung Cancer Working Party has decided that such scheduling is feasible in only a few centres, and that a large multicentre randomised trial comparing alternating against conventional scheduling would be unlikely to achieve a realistic rate of intake. This important scientific comparison has yet to be made.

### References

ARRIAGADA, R., LE CHEVALIER, T., BALDEYROU, P. & 5 others (1985*a*). Alternating radiotherapy and chemotherapy with doxorubicin, etoposide, cyclophosphamide and cisplatin in limited small cell lung cancer. *Cancer Treat. Symp.*, **2,** 115.

ARRIAGADA, R., LE CHEVALIER, T., BALDEYROU, P. & 11 others (1985*b*). Alternating radiotherapy and chemotherapy schedules in small cell lung cancer, limited disease. *Int. J. Radiat. Oncol. Biol. Phys.*, **11,** 1461.

ARRIAGADA, R., PIGNON, J.P. & LE CHEVALIER, T. (1989*a*). Thoracic radiotherapy in small cell lung cancer: rationale for timing and fractionation. *Lung Cancer*, **5,** 237.

ARRIAGADA, R., DE THE, H., LE CHEVALIER, T. & 7 others (1989*b*). Limited small-cell lung cancer: possible prognostic impact of initial chemotherapy doses. *Bull. Cancer*, **76,** 605.

BLEEHEN, N.M. (1986). Radiotherapy for small cell lung cancer. *Chest*, **89,** 268S.

BUNN, P.A., LICHTER, A.S., MAKUCH, R.W. & 8 others (1987). Chemotherapy alone or chemotherapy with chest radiation therapy in limited stage small cell lung cancer: a prospective randomized trial. *Ann. Int. Med.*, **106,** 655.

LE CHEVALIER, T., ARRIAGADA, R., BALDEYROU, P. & 7 others (1987). Association chimio-radiotherapique dans les carcinomes bronchiques à petites cellules. Limites et resultats chez 109 á malades traites avec un schema d'alternance. *Bull. Cancer*, **74,** 559.

LEONARD, R.C.F. (1989). Guest editorial: small cell lung cancer. *Br. J. Cancer*, **59,** 487.

RAWSON, N.S.B. & PETO, J. (1990). An overview of prognostic factors in small-cell lung cancer: a report from the Subcommittee for the Management of Lung Cancer of the United Kingdom Coordinating Committee on Cancer Research. *Br. J. Cancer*, **61,** 597.

SEIFTER, E.J. & IHDE, D.C. (1988). Therapy of small cell lung cancer: a perspective on two decades of clinical research. *Sem. Oncol.*, **15,** 278.

WORLD HEALTH ORGANIZATION (1979). WHO Handbook for Reporting Results of Cancer Treatment. *WHO Offset Publication* No. 48. WHO: Geneva.

WORLD HEALTH ORGANIZATION (1981). *International histological classification of tumours* No. 1: Histological typing of lung tumours, 2nd edition. World Health Organization: Geneva.